# A streamlined, machine learning-derived approach to risk-stratification in heart failure patients with secondary tricuspid regurgitation

**DOI:** 10.1093/ehjci/jead009

**Published:** 2023-02-10

**Authors:** Gregor Heitzinger, Georg Spinka, Sophia Koschatko, Clemens Baumgartner, Varius Dannenberg, Kseniya Halavina, Katharina Mascherbauer, Christian Nitsche, Caroliná Dona, Matthias Koschutnik, Andreas Kammerlander, Max-Paul Winter, Guido Strunk, Noemi Pavo, Stefan Kastl, Martin Hülsmann, Raphael Rosenhek, Christian Hengstenberg, Philipp E Bartko, Georg Goliasch

**Affiliations:** Department of Internal Medicine II, Medical University of Vienna, Währinger Gürtel 18-20, 1090 Vienna, Austria; Department of Internal Medicine II, Medical University of Vienna, Währinger Gürtel 18-20, 1090 Vienna, Austria; Department of Internal Medicine II, Medical University of Vienna, Währinger Gürtel 18-20, 1090 Vienna, Austria; Department of Internal Medicine III, Medical University of Vienna, Währinger Gürtel 18-20, 1090 Vienna, Austria; Department of Internal Medicine II, Medical University of Vienna, Währinger Gürtel 18-20, 1090 Vienna, Austria; Department of Internal Medicine II, Medical University of Vienna, Währinger Gürtel 18-20, 1090 Vienna, Austria; Department of Internal Medicine II, Medical University of Vienna, Währinger Gürtel 18-20, 1090 Vienna, Austria; Department of Internal Medicine II, Medical University of Vienna, Währinger Gürtel 18-20, 1090 Vienna, Austria; Department of Internal Medicine II, Medical University of Vienna, Währinger Gürtel 18-20, 1090 Vienna, Austria; Department of Internal Medicine II, Medical University of Vienna, Währinger Gürtel 18-20, 1090 Vienna, Austria; Department of Internal Medicine II, Medical University of Vienna, Währinger Gürtel 18-20, 1090 Vienna, Austria; Department of Internal Medicine II, Medical University of Vienna, Währinger Gürtel 18-20, 1090 Vienna, Austria; Complexity-Research, Schönbrunner Str. 32 / 20A, 1050 Vienna, Austria; Department of Internal Medicine II, Medical University of Vienna, Währinger Gürtel 18-20, 1090 Vienna, Austria; Department of Internal Medicine II, Medical University of Vienna, Währinger Gürtel 18-20, 1090 Vienna, Austria; Department of Internal Medicine II, Medical University of Vienna, Währinger Gürtel 18-20, 1090 Vienna, Austria; Department of Internal Medicine II, Medical University of Vienna, Währinger Gürtel 18-20, 1090 Vienna, Austria; Department of Internal Medicine II, Medical University of Vienna, Währinger Gürtel 18-20, 1090 Vienna, Austria; Department of Internal Medicine II, Medical University of Vienna, Währinger Gürtel 18-20, 1090 Vienna, Austria; Department of Internal Medicine II, Medical University of Vienna, Währinger Gürtel 18-20, 1090 Vienna, Austria; Herzzentrum Währing, Theresiengasse 43, 1180 Vienna, Austria

**Keywords:** HFrEF, HFpEF, HFmrEF, secondary tricuspid regurgitation, machine learning

## Abstract

**Aims:**

Secondary tricuspid regurgitation (sTR) is the most frequent valvular heart disease and has a significant impact on mortality. A high burden of comorbidities often worsens the already dismal prognosis of sTR, while tricuspid interventions remain underused and initiated too late. The aim was to examine the most powerful predictors of all-cause mortality in moderate and severe sTR using machine learning techniques and to provide a streamlined approach to risk-stratification using readily available clinical, echocardiographic and laboratory parameters.

**Methods and results:**

This large-scale, long-term observational study included 3359 moderate and 1509 severe sTR patients encompassing the entire heart failure spectrum (preserved, mid-range and reduced ejection fraction). A random survival forest was applied to investigate the most important predictors and group patients according to their number of adverse features.

The identified predictors and thresholds, that were associated with significantly worse mortality were lower glomerular filtration rate (<60 mL/min/1.73m^2^), higher NT-proBNP, increased high sensitivity C-reactive protein, serum albumin < 40 g/L and hemoglobin < 13 g/dL. Additionally, grouping patients according to the number of adverse features yielded important prognostic information, as patients with 4 or 5 adverse features had a fourfold risk increase in moderate sTR [4.81(3.56–6.50) HR 95%CI, *P* < 0.001] and fivefold risk increase in severe sTR [5.33 (3.28–8.66) HR 95%CI, *P* < 0.001].

**Conclusion:**

This study presents a streamlined, machine learning-derived and internally validated approach to risk-stratification in patients with moderate and severe sTR, that adds important prognostic information to aid clinical-decision-making.

## Graphical Abstract

**Graphical Abstract:**
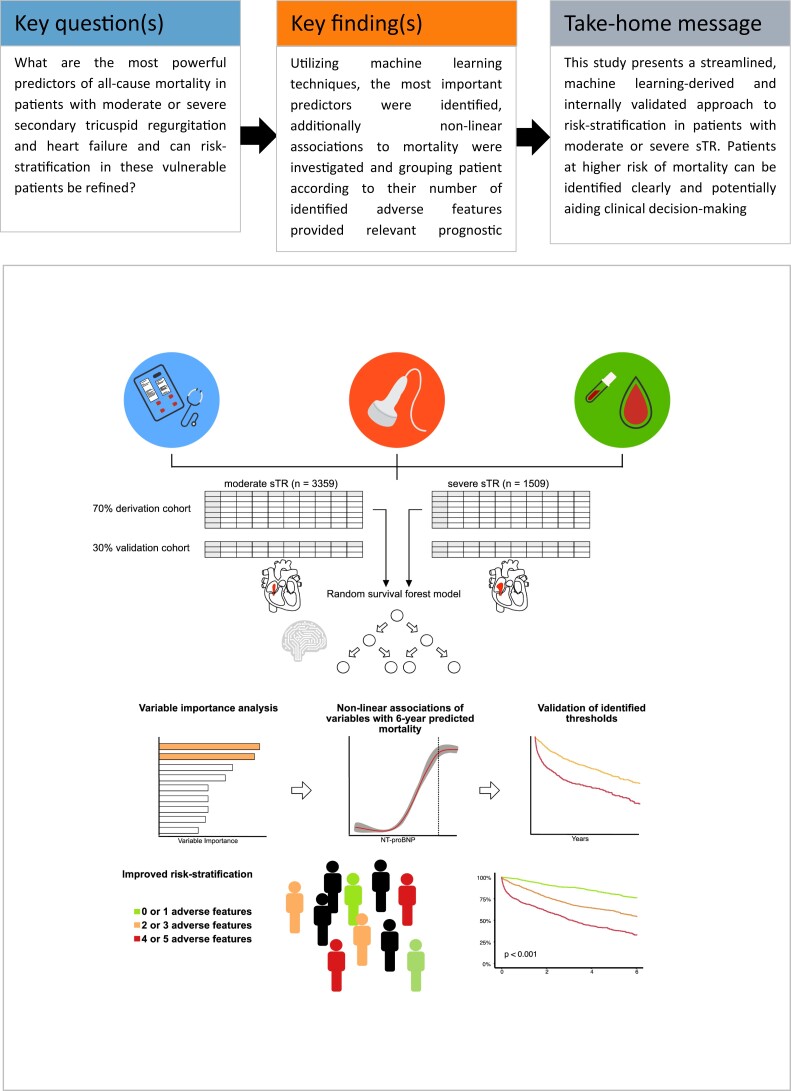

## Introduction

Secondary tricuspid regurgitation (sTR) has been described as the most frequent valvular heart disease, its prevalence rising with advancing age.^1–3^ Significant lesion severity is independently associated with increased morality.^4,5^ Other organ involvements, in particular pulmonary,^6^ hepatic disease^7^ and concomitant left-sided valvular heart disease^8^ are common in sTR,^1^ reflective of advanced cardiovascular and non-cardiovascular disease. This high burden of comorbidities and absence of established outcome benefits may in part explain, why tricuspid interventions are rarely used and often utilized late—when cardiac disease progression is advanced.^9^ Although echocardiographic studies have refined the prognostic impact of significant lesion severity,^4,10^ an integrated approach incorporating clinical comorbidities and laboratory measures for improved risk-stratification is still lacking. Specific scores that focus on estimating the short-term mortality for patients with end-stage liver disease have been successfully applied in patients undergoing tricuspid valve (TV) surgery.^11^ Similarly, a score to predict in-hospital mortality after isolated TV surgery (TRI-SCORE) has been proposed recently.^12^ However, no clinical and echocardiographic parameters are included, and general applicability is limited as TV repair candidates differ from heart failure cohorts.

In recent years, machine learning techniques and data-driven approaches have become increasingly utilized to investigate complex data structures and provide important insights, that are undetectable with conventional statistical methods.^13–17^ Additionally, machine learning is often used for variable importance analysis, feature extraction and investigation of variable interactions, which contribute to refined data analysis.

The aim of this large-scale observational study was to investigate the association between a well-defined and readily available set of clinical, echocardiographic and laboratory variables and all-cause mortality in patients with moderate or severe sTR. Furthermore, the objective was to assess the most powerful predictors and examine potential non-linear associations utilizing machine learning techniques. In the last part of this analysis, we investigated whether the inclusion of identified variables and thresholds would improve the prognostic capability.

## Methods

In this observational cohort study, the Medical University of Vienna health records and echocardiographic database were used to identify all individuals with moderate or severe secondary tricuspid regurgitation and chronic heart failure were included between 2010 and 2020. Heart failure was diagnosed according to current guidelines and categorized into heart failure with preserved ejection fraction [HFpEF, left ventricular ejection fraction (LVEF) ≥ 50%], heart failure with mildly reduced ejection fraction (HFmrEF, LVEF 41–49%), and heart failure with reduced ejection fraction (HFrEF, LVEF ≤ 40%).^18^ This methodology enabled full compliance with the guideline definitions of heart failure, which are different for the three ejection fraction ranges and include mandatory features for a correct diagnosis, such as relevant structural heart disease, diastolic dysfunction, signs and symptoms, and raised levels of natriuretic peptides. Clinical, echocardiographic and laboratory data were collected at the time of the echocardiographic exam with a tolerance of ± 7 days. Patients with primary TV disease (stenotic, or regurgitant) were excluded. Moreover, we did not include individuals with significant (more, or equal to moderate) aortic valve, pulmonary valve, or primary mitral valve disease. Primary outcome was all-cause mortality, obtained by retrieval query from the national death registry.

## Study population

### Derivation and validation datasets

The total study cohort was split according to sTR severity and within each severity group patients were allocated to either a derivation, or validation cohort. Patients were split by random sampling under two conditions (see Supplementary material online, *Table S1* for R package implementation): (i) 70% of each dataset was used as a derivation cohort and the remaining 30% was used as validation data. (ii) The splitting procedure was stratified by HF subtype to provide a similar subgroup representation as in the original dataset.

### Echocardiography

All echocardiographic exams were recorded according to a structured standard protocol and read by board-certified physicians as recommended by current guidelines.^19,20^ Each patient underwent a comprehensive and complete transthoracic echocardiographic exam—focused, incomplete, and examinations with insufficient data quality were excluded. Standard four- and two-chamber views were utilized to assess cardiac morphology by the left and right ventricular diameters. Atrial dimension were assessed from four-chamber views by long axis length, or volume. Left ventricular systolic function was calculated using the biplane Simpson method and graded according to recommended ejection fraction cut-offs(HFpEF ≥ 50%, HFmrEF 41–49%, and HFrEF ≤ 40%). Mitral regurgitation severity was assessed by an integrated approach as previously described,^21^ comprising mitral valve morphology, width of the proximal regurgitant jet, proximal flow convergence, and pulmonary venous flow pattern. STR was graded by an integrative approach as recommended.^20^ Systolic pulmonary artery pressure was calculated by addition of the peak tricuspid regurgitation systolic gradient to the estimated central venous pressure.

### Statistical analysis

A detailed report of the statistical analysis is presented in the supplemental material. In brief, continuous baseline data are presented as the median and interquartile range (IQR) and categorical data by count and percent. Kruskal-Wallis test and chi-square test were used accordingly for comparison. All analyses were conducted with the derivation cohorts and the proportion of missing values was small. A random survival forest (RSF) analysis (detailed description in supplement) for all-cause mortality and variable importance was performed. Results were validated and further analysed by Kaplan-Meier survival and Cox regression analysis. Univariable Cox regression was performed with each predictor and adjusted for common clinical risk factors. All patients were summarized in distinct groups according to their number of adverse features. The *Graphical Abstract* provides a visual reproduction of the methodology used. The outlined methodology was planned according to the Proposed Requirements for Cardiovascular Imaging-Related Machine Learning Evaluation (PRIME) guidelines (see Supplementary material online, *Table S2)*.^22^ All analyses assumed a two-sided *P*-value <0.05 to be statistically significant. The R software (R Core Team (2020). R: A language and environment for statistical computing. R Foundation for Statistical Computing, Vienna, Austria. URL https://www.R-project.org/.) was used for all analyses. A detailed description of packages and their usage is provided *in*Supplementary material online, *Table S1*.

## Results

### Baseline characteristics of the total study population

A total of 4868 patients [median age 74 (66–80)] with moderate (*n* = 3359) and severe (*n* = 1509) sTR were included in this observational study, 58% of which were male. 52% were allocated to the HFpEF subtype, 22% had HFmrEF and 26% were diagnosed with HFrEF. A detailed overview of baseline characteristics according to sTR severity is presented in *Table 1* and Supplementary material online, *Table S3*. Comorbidity burden was high in both severity groups, 47% in the moderate sTR group and 42% in the severe sTR group had a history of coronary artery disease (CAD). Similarly, hypertension and atrial fibrillation (AF) were also frequent in both severity groups. Right ventricular end-diastolic diameter (RVEDD) and measurements of atrial dimension were larger in severe sTR patients (RVEDD 35 mm [31–39] vs. 40 mm [35–44]) (*P* < 0.001), left atrial (LA) diameter 61 mm [56–67] vs. 65 mm [60–71] (*P* < 0.001) and right atrial diameter (RA) 60 mm [55–65] vs. 66 mm [60–72] (*P* < 0.001). Severe left ventricular (LV) dysfunction was present in 1273 (26%) patients and 978 (6.4%) had severe right ventricular (RV) impairment. Of note, severe RV dysfunction was more frequent in patients with severe sTR (12% vs. 3.8%, *P* < 0.001). Pacemaker leads were more frequent in severe sTR (312 [21%] vs. 455 [14%], *P* < 0.001) (*Table 1*) and more common in HFrEF patients (HFrEF 333 [26%] vs. HFmrEF 188 [18%] vs. HFpEF 246 [9.7%], *P* < 0.001).

**Table 1:** Baseline clinical, echocardiographic and laboratory parameters of patients with moderate and severe secondary tricuspid regurgitation

Characteristic	Overall, *n* = 4868^a^	moderate sTR, *n* = 3359^a^	severe sTR, *n* = 1509^a^	*P*-value^b^
***Clinical characteristics***				
Heart failure subtype				**<0**.**001**
ȃHFpEF	2542 (52%)	1832 (55%)	710 (47%)	
ȃHFmrEF	1053 (22%)	748 (22%)	305 (20%)	
ȃHFrEF	1273 (26%)	779 (23%)	494 (33%)	
Sex, male	2845 (58%)	1997 (59%)	848 (56%)	**0**.**033**
Age, years	74 (66–80)	73 (66–80)	74 (66–81)	**0**.**019**
Body mass index, kg/m^2^	26.3 (23.7–29.4)	26.6 (24.2–29.4)	25.3 (22.5–29.4)	**<0**.**001**
Weight	78 (67–88)	79 (69–89)	74 (65–87)	**<0**.**001**
Height	170 (163–177)	170 (163–177)	170 (163–176)	0.13
***Comorbidities***				
Hypertension	2895 (59%)	2094 (62%)	801 (53%)	**<0**.**001**
Hyperlipidemia	1477 (30%)	1084 (32%)	393 (26%)	**<0**.**001**
Diabetes, type II	1144 (24%)	812 (24%)	332 (22%)	0.10
Coronary Artery Disease	2207 (45%)	1572 (47%)	635 (42%)	**0**.**002**
Atrial fibrillation	2105 (43%)	1370 (41%)	735 (49%)	**<0**.**001**
COPD^c^	717 (15%)	468 (14%)	249 (17%)	**0**.**019**
Cerebral vascular disease	918 (19%)	660 (20%)	258 (17%)	**0**.**035**
Peripheral vascular disease	1156 (24%)	806 (24%)	350 (23%)	0.5
***Echocardiographic parameters***				
Left ventricular end-diastolic diameter, mm	47 (42–52)	47 (43–52)	47 (42–53)	0.5
Left atrial diameter, mm	62 (57–68)	61 (56–67)	65 (60–71)	**<0**.**001**
Right ventricular end-diastolic diameter, mm	36 (32–41)	35 (31–39)	40 (35–44)	**<0**.**001**
Right atrial diameter, mm	61 (56–68)	60 (55–65)	66 (60–72)	**<0**.**001**
Left ventricular dysfunction				**<0**.**001**
ȃAbsent	2325 (48%)	1674 (50%)	651 (43%)	
ȃMild	663 (14%)	475 (14%)	188 (12%)	
ȃModerate	607 (12%)	431 (13%)	176 (12%)	
ȃSevere	1273 (26%)	779 (23%)	494 (33%)	
Right ventricular dysfunction				**<0**.**001**
ȃMild	3312 (68%)	2594 (77%)	718 (48%)	
ȃModerate	1244 (26%)	639 (19%)	605 (40%)	
ȃSevere	312 (6.4%)	126 (3.8%)	186 (12%)	
Secondary mitral regurgitation				**<0**.**001**
ȃMild	591 (12%)	457 (14%)	134 (8.9%)	
ȃModerate	3299 (68%)	2414 (72%)	885 (59%)	
ȃSevere	978 (20%)	488 (15%)	490 (32%)	
Pulmonary artery pressure (mmHg)	51 (44–62)	48 (41–59)	56 (48–69)	**<0**.**001**
PM^e^ leads present	767 (16%)	455 (14%)	312 (21%)	**<0**.**001**
Inferior vena cava diameter, mm	22.0 (19.0–25.0)	21.0 (18.0–24.0)	24.0 (20.0–27.0)	**<0**.**001**
Nt-proBNP, pg/mL	2651.2 (1072.8–6010.1)	2250.0 (870.9–5022.5)	3725.0 (1720.0–8163.0)	<0.001

*n* (%); Median (IQR).

Pearson's Chi-squared test; Wilcoxon rank sum test; bold *P* values indicate statistical significance.

Chornic obstructive pulmonary disease.

High sensitivity C-reactive protein.

Pacemaker.

### Derivation and validation cohort baseline characteristics

Baseline characteristics according to derivation/validation cohort for each severity group are presented in Supplementary material online, *Tables S4 and S5*. In the severe sTR cohort, a total of 1057 patients served as the derivation cohort with the remaining 452 as validation. HF subgroups were equally weighted in both datasets. In regard to moderate sTR patients, conditional random sampling assigned 2353 patients to the derivation group and the remaining 1006 were allocated to the validation set. Baseline characteristics according to the derivation/validation cohort showed equal distribution of clinical, echocardiographic and laboratory features and no evidence of systemic bias.

### Importance of variables in the RSF models

**Figure 1:**
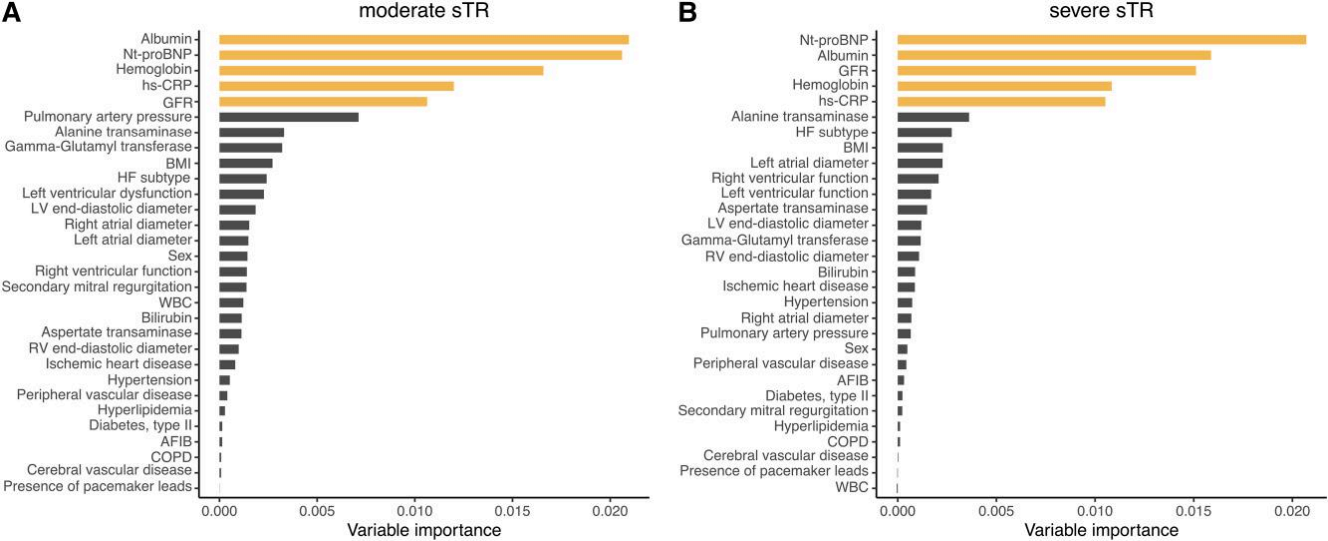
Variable importance analysis in moderate (*A*) and severe sTR (*B*) patients. Investigation of variable importance in two random survival forest models, encompassing a broad spectrum of clinical, echocardiographic and laboratory variables. The five most predictive variables consisted of serum albumin, Nt-proBNP, hemoglobin, glomerular filtration rate and C-reactive protein. AFIB = atrial fibrillation, BMI = body mass index, COPD = chronic obstructive pulmonary disease, hs-CRP = high sensitivity C-reactive protein, HF = heart failure, GFR = glomerular filtration rate, WBC = white blood cell count.

Both RSF models were constructed with a total of 30 variables, comprising of clinical, echocardiographic and laboratory predictors using the respective derivation cohorts. Variable importance for each severity model is depicted in *Figure 1*. In both sTR severity groups laboratory parameters were the most important predictors of all-cause mortality. In moderate and severe sTR patients, serum albumin and NT-proBNP levels were considered the most powerful predictors. Interestingly, common clinical risk factors such as atrial fibrillation and ischaemic heart disease offered comparably low predictive power in those specific models and consistently ranked within the lower spectrum of variable importance. Regarding echocardiographic variables, systolic pulmonary artery pressure (sPAP) was ranked the highest in moderate sTR patients and HF subtype in severe sTR.

### Dependency and partial dependency of selected variables in moderate and severe sTR

**Figure 2:**
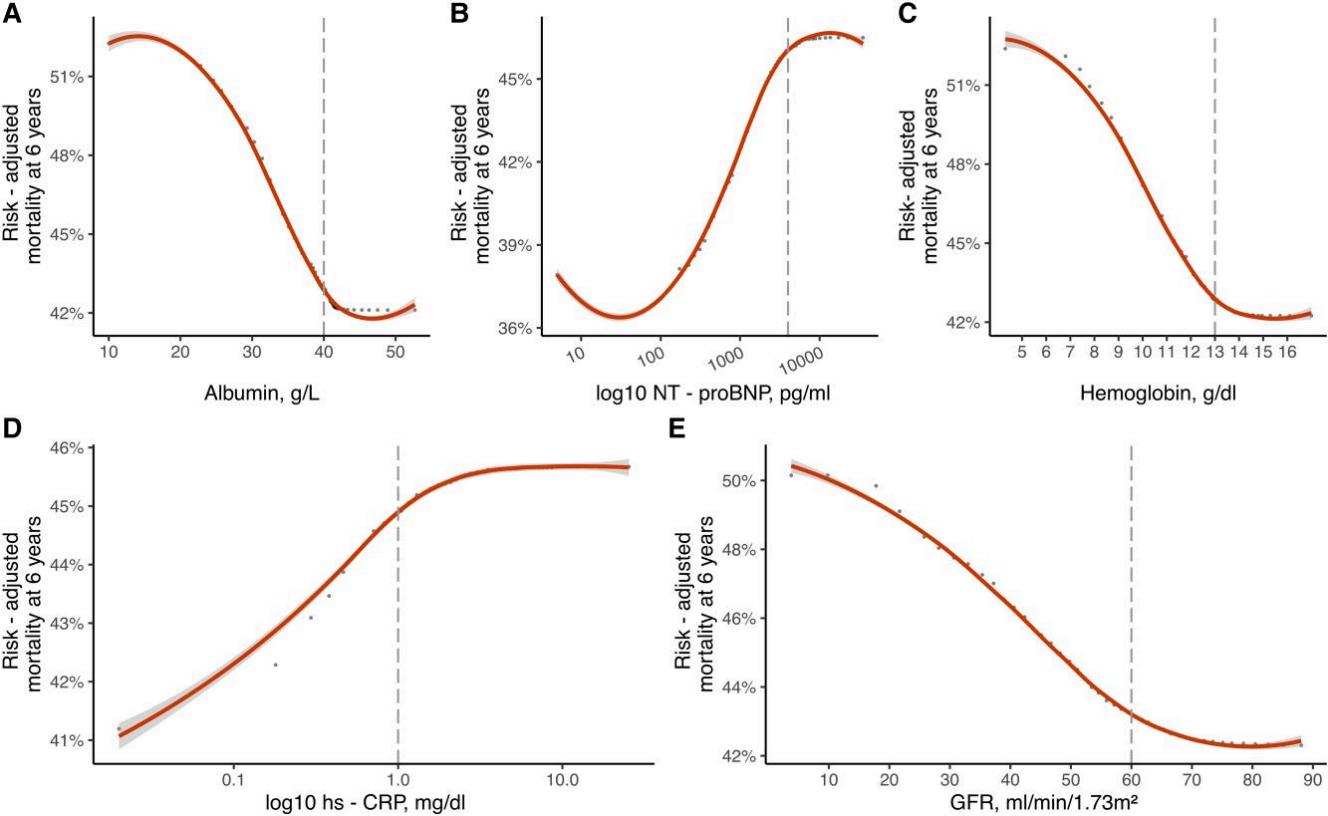
Partial dependency plots in moderate sTR RSF model. Partial dependency plots of the five most predictive variables (*A–E*) to investigate potential non-linear associations with risk-adjusted mortality at 6 years in patients with moderate sTR. Optimal thresholds for each parameter are denoted by dashed line. Hs-CRP = high sensitivity C-reactive protein, GFR = glomerular filtration rate.

**Figure 3:**
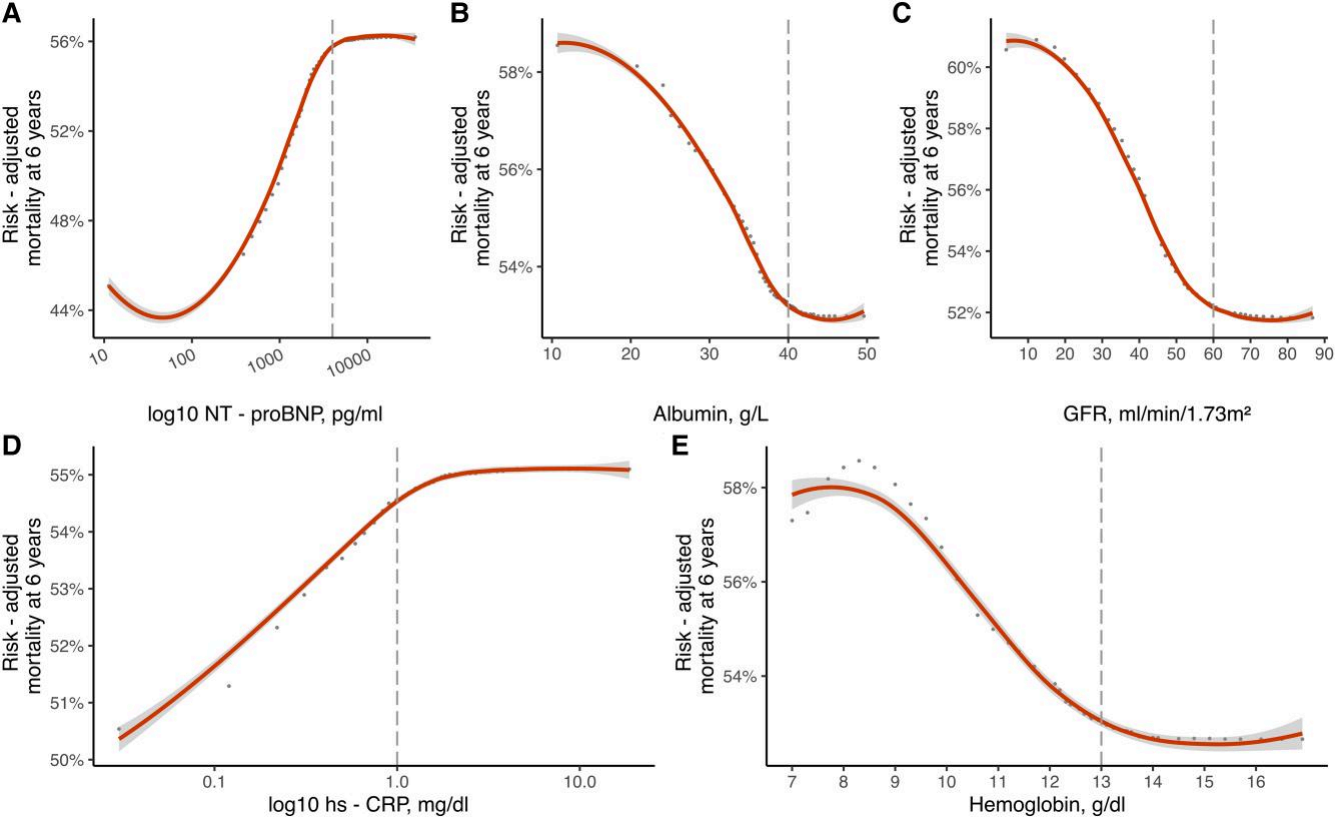
Partial dependency plots in severe sTR RSF model. Partial dependency plots of the five most predictive variables (*A–E*) to investigate potential non-linear associations with risk-adjusted mortality at 6 years in patients with severe sTR. Optimal thresholds for each parameter are denoted by dashed line. Hs-CRP = high sensitivity C-reactive protein, GFR = glomerular filtration rate.

Dependency plots from the derivation cohorts for moderate and severe sTR are presented in *Supplementary Figures S1 and S2*. In brief, the predicted mortality at 6 years increased continuously with rising age and high-sensitivity C-reactive protein (hs-CRP) on a logarithmic scale. Albumin, glomerular filtration rate (GFR) and hemoglobin both showed an inverse relationship with mortality. A similar pattern can be observed in severe sTR patients. The non-adjusted association of log-10 NT-proBNP with mortality increased strongly for log-10 Nt-proBNP values below 4000 pg/mL in severe sTR and continued to rise with increased neurohumoral activation, albeit the effect was not as pronounced (see Supplementary material online, *Figure S2, Plot A*). Partial dependency plots of the five most important predictors for each severity cohort are presented in *Figures 2* and *3*. These demonstrate the adjusted variable dependency on mortality, when the effects of all other variables are averaged out. In both moderate and severe sTR patients the serum albumin levels below 40 g/L resulted in drastic mortality increases. Patients with NT-proBNP levels of < 100 pg/mL had the best survival, risk of mortality increased continuously with higher NT-proBNP levels, but plateaued after 4000 pg/mL. Similarly, hs-CRP levels ≥ 1.0 mg/dL were associated with an elevated (*Figure 2*, *Plot D* and *Figure 3 Plot D*), but stable risk of mortality at 6 years. In contrast, hs-CRP levels < 1.0 mg/dL were at significantly lower risk of mortality (see Supplementary material online, *Figure S5* and *S6*, *Plot G* and *H*; *Table 2*). Histograms of variable distributions used for threshold identification are presented in Supplementary material online, *Figures S3* and *S4*.

**Table 2:** Univariable and adjusted cox analysis for validation cohort

Moderate sTR	Validation (*n* = 1006)	
	Hazard ratio (95% CI)	*P*-value ^a^
**Univariable Cox analysis**		
Albumin ≥ 40 g/L	0.38 (0.30–0.47)	**<0**.**001**
NT-proBNP ≥ 4000 pg/mL	2.48 (2.02–3.03)	**<0**.**001**
Hemoglobin ≥ 13 g/dL	0.45 (0.36–0.56)	**<0**.**001**
Hs-CRP^c^ ≥ 1.0 mg/dL	2.03 (1.65–2.48)	**<0**.**001**
GFR ≥ 60 mL/min/1.73m^2^	0.65 (0.53–0.79)	**<0**.**001**
**Clinical risk factor adjustd Cox analysis** ^b^		
Albumin ≥ 40 g/L	0.39 (0.31–0.50)	**<0**.**001**
NT-proBNP ≥ 4000 pg/mL	2.58 (2.02–3.30)	**<0**.**001**
Hemoglobin ≥ 13 g/dL	0.49 (0.39–0.61)	**<0**.**001**
Hs-CRP^c^ ≥ 1.0 mg/dL	1.96 (1.59–2.41)	**<0**.**001**
GFR ≥ 60 mL/min/1.73m^2^	0.69 (0.55–0.86)	**<0**.**001**
**Severe sTR**	**Validation (*n* = 452)**	
**Univariable Cox analysis**		
Albumin ≥ 40 g/L	0.42 (0.31–0.58)	**<0**.**001**
NT-proBNP ≥ 4000 pg/mL	2.65 (2.01–3.50)	**<0**.**001**
Hemoglobin ≥ 13 g/dL	0.49 (0.36–0.66)	**<0**.**001**
Hs-CRP^c^ ≥ 1.0 mg/dL	1.98 (1.50–2.60)	**<0**.**001**
GFR ≥ 60 mL/min/1.73m^2^	0.55 (0.41–0.73)	**<0**.**001**
**Clinical risk factor adjustd Cox analysis** ^b^		
Albumin ≥ 40 g/L	0.48 (0.35–0.67)	**<0**.**001**
NT-proBNP ≥ 4000 pg/mL	2.15 (1.56–2.95)	**<0**.**001**
Hemoglobin ≥ 13 g/dL	0.53 (0.38–0.73)	**<0**.**001**
Hs-CRP^c^ ≥ 1.0 mg/dL	1.79 (1.35–2.38)	**<0**.**001**
GFR ≥ 60 mL/min/1.73m^2^	0.65 (0.48–0.90)	**0**.**009**

Bold *P* values indicate statistical significance.

Adjusted for sex, age, history of ischaemic heart disease, serum creatinine, LV end-diastolic diameter, left ventricular function and right ventricular function.

High sensitivity C-reactive protein.

### Validation of machine learning derived cut-offs

All identified cut-offs were then further investigated to verify the clinical relevancy and applicability. Kaplan-Meier analysis with all RSF-derived thresholds were performed for moderate (see Supplementary material online, *Figure S5*) and for severe sTR patients (see Supplementary material online, *Figure S6*) and yielded similar results for both derivation and validation cohorts. Differentiation of identified cut-offs were significant in all predictors. Cox regression analysis was performed in an univariable model for each threshold and also adjusted for common clinical confounders. In both patients with severe and moderate sTR, higher albumin levels (univariable moderate sTR: Albumin ≥40 g/L 0.38 [0.30–0.47] hazard ratio (HR) and 95% confidence intervals (CI), *P* < 0.001, univariable severe sTR: Albumin ≥40 g/L 0.42 [0.31–0.58] HR 95% CI, *P* < 0.001), higher hemoglobin levels (univariable moderate sTR: hemoglobin ≥ 13 g/dL 0.45 [0.36–0.56] HR 95% CI, *P* < 0.001, univariable severe sTR: hemoglobin ≥ 13 g/dL 0.49 [0.36–0.66] HR 95% CI, *P* < 0.001), lower hs-CRP levels (univariable moderate sTR: hs-CRP ≥1.0 mg/dL 2.03 [1.65–2.48] HR 95% CI, *P* < 0.001, univariable severe sTR: hs-CRP ≥1.0 mg/dL 1.98 [1.50–2.60] 95% CI, *P* < 0.001) and higher GFR levels (univariable moderate sTR: GFR ≥60 mL/min/1.73m^2^ 0.65 [0.53–0.79] HR 95% CI, *P* < 0.001, univariable severe sTR: GFR ≥60 mL/min/1.73m^2^ 0.55 [0.41–0.73] 95% CI, *P* < 0.001) were associated with favourable survival. Results remained similar in univariable and adjusted Cox regression and were significant for both derivation and validation cohort (*Table 2* and Supplementary material online, *Table S6*).

### Incremental prognostic information of machine learning derived thresholds

**Figure 4:**
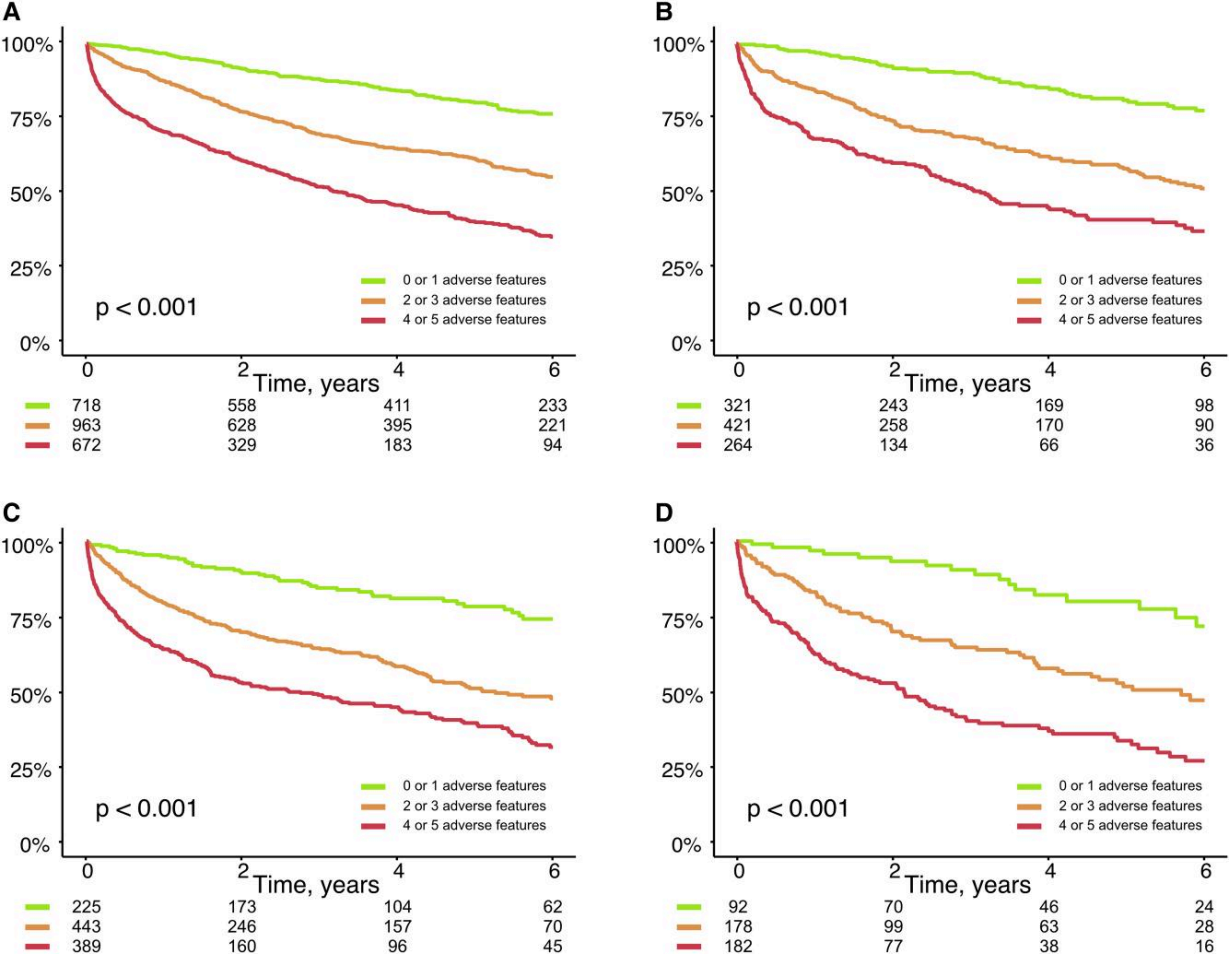
Prognostic impact according to the number of adverse features. The most important predictors were identified and patients stratified according to their number of adverse features. Kaplan-Meier analysis shows excellent risk-stratification in patients with moderate sTR (derivation cohort *Figure 4/Plot A* and validation cohort *Figure 4/Plot B*) and in severe sTR (derivation cohort *Figure 4/Plot C* and validation cohort *Figure 4/Plot D*).

Additional analyses to assess the incremental prognostic value of machine learning thresholds were performed. Patients were grouped according to the number of adverse thresholds as identified into 3 groups (0 or 1, 2 or 3 and 4, or 5 adverse features). In patients with moderate or severe sTR, adverse features encompassed serum albumin < 40 g/L, NT-proBNP ≥ 4000 pg/mL, hemoglobin < 13 g/dL, hs-CRP ≥ 1.0 mg/dL, and GFR < 60 mL/min/1.73m^2^. Patients with 4 or more adverse features had the worst survival (moderate sTR validation: 4.81 [3.56–6.50] HR 95% CI, *P* < 0.001, severe sTR validation: 5.33 [3.28–8.66] HR 95% CI, *P* < 0.001) as delineated in *Table 3* and Supplementary material online, *Table S7*. Further examination revealed that the addition of adverse features (Model 2) to the baseline clinical risk factor model (Model 1) provides improved predictive value (see Supplementary material online, *Table S8*). C-statistic was higher in both severity groups with the added prognostic information of the number of adverse features. Additionally, further investigation by Kaplan-Meier analysis, depicted in *Figure 4*, revealed that excellent risk-stratification according to the number of adverse features in both moderate and severe sTR groups.

**Table 3:** Cox regression analysis in validation cohort for adverse feature subgroups

Moderate sTR	Validation	
	Hazard ratio (95% CI)	*P*-value^a^
0 or 1 adverse features (Reference)		
2 or 3 adverse features	2.73 (2.03–3.67)	**<0**.**001**
4 or 5 adverse features	4.81 (3.56–6.50)	**<0**.**001**
**Severe sTR**		
0 or 1 adverse features (Reference)		
2 or 3 adverse features	2.64 (1.60–4.37)	**<0**.**001**
4 or 5 adverse features	5.33 (3.28–8.66)	**<0**.**001**

Bold *P* values indicate statistical significance.

## Discussion

This paper presents a unique approach to risk-stratification in patients with moderate and severe sTR utilizing machine learning techniques in a large cohort with readily available clinical, echocardiographic and laboratory parameters, that yields incremental prognostic information. The main findings are (i) identification of the most relevant predictors in moderate and severe sTR, (ii) detection of non-linear associations with mortality at six years, (iii) prognostic thresholds of identified variables to define risk groups, (iv) a heterogenous spectrum of TR patients, that can be streamlined according to the number of adverse features and (v) consistent and robust results upon internal validation, thereby indicating a feasible tool for patient management and risk-stratification.

### sTR and the need for risk-stratification

Significant TV regurgitant lesions are very frequent in an aging population and in particular in heart failure cohorts. Recent reports indicate a prevalence of up to 0.55% in a sex- and age adjusted general US population,^1^ comparable with the prevalence of aortic stenosis.^23^ Moreover, there is ample evidence of high mortality in patients with significant sTR, especially with multiple lesion involvement,^8^ yet treatment of sTR is underused and often initiated too late, when the window of intervention has already closed. The reasons for underutilization of therapeutic options and late referrals remain a subject of further investigation. Both historically unsatisfactory surgical outcomes^24^ and frequently high comorbidity burden of sTR patients with multiple organ involvement are likely to play an essential role in the decision-making process.^25^ Importantly, our data also indicate that severe right ventricular dysfunction nearly tripled in frequency (12% vs. 3.8%, *P* < 0.001) in severe sTR—possibly further influencing therapeutical approaches. However, with recent advances in transcatheter TV interventions (TTVI),^26,27^ the approach to sTR treatment will be subject to comprehensive reevaluations.

This discrepancy in treatment need and intention highlight the importance of improved risk-stratification, that allows for reliable and comprehensive analysis of patients. We therefore propose a refined stratification of sTR patients with machine learning derived variable importance analysis and validated cut-offs utilizing readily available features, that incorporate clinical, echocardiographic and laboratory parameters.

### Variable importance and non-linear associations

One of the many advantages of a machine learning technique application is the ability to assess the importance of each variable in the model, in order to select the most powerful predictors for the outcome. In this case, we investigated both sTR models and found similar results in patients with moderate and severe sTR. Nt-proBNP was ranked as the most important predictor of mortality in severe sTR patients and the second most powerful in moderate sTR, albeit with a marginal difference to serum albumin. This result is in accordance with current literature, as a recent study, investigating 1034 elderly patients with TR found that higher NT-proBNP ratio values yielded significant prognostic information. Of note, only 436 patients had secondary aetiology of TR.^28^ Further investigation using partial dependence plots found the risk-adjusted mortality to be lowest for patients with NT-proBNP values < 100 pg/mL (*Figure 2*, *Plot B* and *Figure 3*, *Plot A*). With increasing neurohumoral activation mortality rose (> 10% increase in severe sTR), but did not further increase after 4000 pg/mL. A previous study has reported on the loss of prognostic power in patients with significant sTR and higher NT-proBNP.^29^ A similar pattern can be observed for hs-CRP. This parameter was ranked among the most predictive in both models and mortality at 6 years increased for values above 1.0 mg/dL, but not thereafter. Markers of inflammation are known predictors of HF and cardiovascular disease,^30^ but the prognostic value of hs-CRP in moderate or severe sTR and HF has not been described yet and warrants further investigation as a potential biomarker in heart failure. Hypoalbuminemia is another common laboratory finding in heart failure patients, that has significant prognostic power.^31–33^ This is supported by the current findings, where both patients with moderate and severe sTR experienced a rapid increase in 6-year mortality for albumin levels below 40 g/L. The aetiology of hypoalbuminemia in HF is complex and multifactorial, common causes include decreased hepatic synthesis,^7^ hemodilution due to fluid overload and malnutrition.^34,35^ In severe and moderate sTR, chronic hepatic congestion may lead to intrinsic liver damage and contribute to hypoalbuminemia.

### Echocardiographic and laboratory parameters

Both RSF models continuously ranked laboratory parameters among the most predictive for the outcome, while echocardiographic parameters surprisingly were graded as mediocre predictors. This is in contrast to previous data that reported significant predictive power of echocardiographic variables.^4,36^ These findings, however, do not detract from the usefulness of echocardiographic parameters, but highlight that in direct comparison in the same model laboratory variables provide more predictive power than echocardiographic parameters in regard to the endpoint of all-cause mortality.

### Number of adverse features and prognostic impact

All machine learning derived predictive variables and identified thresholds proved to differentiate well between patients with high survival rates and patients with high mortality (see Supplementary material online, *Figure S5* and *S6*). Upon application of these thresholds to our validation cohorts, results remained similar and robust. We also demonstrated that grouping patients according to their number of adverse features, provides additive predictive information in comparison to our baseline model with clinical risk factors. This allows for efficient, long-term and internally validated risk-stratification. We hypothesize, that clinical applicability is feasible and supported by the use of common parameters, that are readily available.

A recent consensus paper for the management of secondary mitral regurgitation highlighted, that transcatheter procedures carry significant risk for the individual patient, especially in patients with high burden of comorbidities and limited life-expectancy.^37^ In these situations, a palliative setting should be considered. Although, similar recommendations for tricuspid regurgitation do currently not exist, they nevertheless provide a prudent guide for sTR, due to many similarities. We hypothesize, that improved risk-stratification according to the number of adverse features may aid in challenging scenarios and support evidence-based decision-making.

### Strengths and limitations

Particular strengths of this study are: (i) the large study cohort with long-term outcome data, that allow for sufficiently large derivation and validation cohorts, (ii) accurate HF diagnosis and of all HF subtypes, according to guideline recommendations, (iii) consistent results upon internal validation and (iv) comprehensive and broad inclusion of patients with a high burden of comorbidities and multiple organ involvement. Our results present the most contemporary insights into risk-stratification in patients with secondary TR. Internal validation indicated consistent findings. Patients were included from a high-volume tertiary-care centre, which ensure adherence to a clinical routine and guideline recommendations. However, a centre-specific bias cannot be excluded and therefore external validation would provide additional information of data reliability and applicability in clinical practice. Hemodynamic status including heart rate and blood pressure are lacking and could affect severity assessment at baseline. Right heart catheterization would provide more information regarding pulmonary pressures, as echocardiographic estimation is frequently not accurate in severe sTR. Additionally, more granular data on medications and dosage was not available but could provide further insights into heart failure management in the setting of sTR.

## Conclusion

This study for the first time presents a streamlined, machine learning-derived approach to risk-stratification in patients with moderate and severe secondary tricuspid regurgitation. A high comorbidity burden is frequent in sTR patients and complicates patient management. The inclusion of readily available clinical, echocardiographic and laboratory parameters cover a broad spectrum of disease. Patients with lower hemoglobin, worse renal function, more inflammation, low serum albumin and higher neurohumoral activation are at high risk of mortality independent of sTR severity. This data-driven approach adds important prognostic information. Results are internally validated and aim to provide support in clinical-decision-making.

## Supplementary material

Supplementary materials are available at *European Heart Journal - Cardiovascular Imaging* online.

## Supplementary Material

jead009_Supplementary_Data

## Data Availability

Due to patient safety, access to individual participant data is restricted and datasets are not publicly available at the current time. Deidentified data for this study can be made available upon request to the corresponding author with a data sharing agreement.

## Abbreviations

HFmrEF: heart failure with mid-range ejection fraction (LVEF 40–49%)
HFpEF: heart failure with preserved ejection fraction (LVEF ≥ 50%)
HFrEF: heart failure with reduced ejection fraction (LVEF < 40%)
HF: heart failure
LV: left ventricular
LVEF: left ventricular ejection fraction
STR: secondary tricuspid regurgitation
TV: Tricuspid valve
