# Supplementary material for: A streamlined, machine learning-derived approach to risk-stratification in heart failure patients with secondary tricuspid regurgitation
Source: Eur Heart J Cardiovasc Imaging. 2023 Feb 10;24(5):588–97. doi: 10.1093/ehjci/jead009 (PMC10125224; doi:10.1093/ehjci/jead009)
Supplement: jead009_Supplementary_Data [file jead009_supplementary_data.docx]

**Supplement for: A streamlined, machine learning-derived approach to risk-stratification in heart failure patients with secondary tricuspid regurgitation.** by Heitzinger et al.

**Statistical analysis**

Continuous baseline data are presented as median and interquartile range (IQR) and categorical data by count and percent. Kruskal-Wallis test and chi-square test were used accordingly for comparison. All analyses were conducted with the derivation cohort for each sTR severity grade. The proportion of missing values was small and these were imputed with the *missForest* algorithm (*Supplementary Table1*) before the main analysis ^1^. To identify the most important predictors of mortality in both moderate and severe sTR, a random survival forest (RSF) analysis for all-cause mortality was performed in each severity grade to identify the most important variables and investigate non-linear associations. Nonlinear associations and derived thresholds were then validated on the respective validation datasets and further analyzed by Kaplan-Meier survival and Cox regression analysis. Proportionality assumptions were investigated using Schoenfeld residuals. Univariable Cox regression was performed with each identified cut-off and additionally adjusted for common clinical risk factors, encompassing age, gender, ischemic heart disease, serum creatinine levels, left ventricular end-diastolic diameter, left ventricular and right ventricular function. All patients were than summarized in distinct groups according to their number of adverse features. All cutoffs and regression analysis were then internally validated on the two datasets. The *Graphical abstract* provides a visual reproduction of the methodology used. Lastly, patients were grouped according to the number of adverse features and the prognostic impact was assessed using C-statistics and Kaplan-Meier curves. The outlined methodology was planned according to the recommendations from the Proposed Requirements for Cardiovascular Imaging-Related Machine Learning Evaluation (PRIME) guidelines. A checklist can be found in *Supplementary Table 2* ^2^. All analyses assumed a two-sided p-value <0.05 to be statistically significant. The R software (R Core Team (2020). R: A language and environment for statistical computing. R Foundation for Statistical Computing, Vienna, Austria. URL https://www.R-project.org/.) was used for all analyses. A detailed description of packages and their usage is provided *in Supplementary Table 1*.

**Random survival forest analysis**

This type of machine learning is a special form of random forest analysis for right censored data ^3^. A random forest is an ensemble method of a binary decision tree. Each tree is grown from a root node and is then recursively split into daughter nodes to maximize survival difference utilizing log-rank statistics ^4^. At each split $\sqrt{p}$ (where p is the total number of predictors) random predictors are considered as a splitting variable. For optimization several varying number of trees were used, but in the final RSF analysis 1000 trees were grown for each sTR severity grade. After RSF model development, predictiveness for each variable was assessed by their relative variable importance, which uses a prediction error approach. Here the change in prediction error is analyzed as each variable is randomly permuted (termed ‘noised up’). Negative or close to zero values indicate little contribution to the predictive ability. The five most important predictors for each severity grade where then further investigated. Dependency and partial dependency plots were used to evaluate potential nonlinear associations. Dependency plots delineate the unadjusted overall trend of the predicted mortality in relation to the variable of interest. Partial dependency plots on the other hand display the risk-adjusted association of a variable on mortality. They are constructed by averaging out the effects of all other variables in the RSF model and therefore only depict the effect of the variable under investigation. In case of nonlinear associations, thresholds were derived considering the nadir, histograms of value distribution and normal range for the variable.

**Supplementary Table 1:** R packages used for data analysis and corresponding version.

| **Package** | **Version** | **Use** |
| --- | --- | --- |
| tidyverse | 1.3.1 | Data analysis, general data handling/plotting/ programming/ import/ data subsampling |
| rsample | 0.0.8 | Random sampling split with stratification/initial_split() function |
| gtsummary | 1.4.2 | Summary of baseline characteristics/ table generation |
| ggplot2 | 3.35 | Plot generation |
| missForest | 1.4 | Random forest algorithm for data imputation |
| tidymodels | 0.1.2 | Dataset splitting into derivation/validation cohort |
| survival | 3.2-11 | Survival analysis/ Cox regression |
| randomForestSRC | 2.12.0 | Random survival Forest analysis |
| ggRandomForests | 2.0.1 | Dependency and Partial dependency plots |
| survminer | 0.4.8 | Kaplan – Meier- analysis |

**Supplementary Table 2**:ML checklist according to PRIME Guidelines

| **Section** | **Checklist item** | **Application to this paper** |
| --- | --- | --- |
| 1.1 | Describe the need for the application of machine learning to the dataset | Introduction, to provide a refined risk-stratification, delineate non-linear associations |
| 1.2 | Describe the objectives of the machine learning analysis | Introduction, to provide a comprehensive risk-stratification, investigate variable importance and non-linear associations |
| 1.3 | Define the study plan | Introduction and Methods/statistical analysis |
| 1.4 | Describe the summary statistics of baseline data | Table 1 |
| 1.5 | Describe the overall steps of the machine learning workflow | Graphical abstract and Methods/statistical analysis |
| 2.1 | Describe how the data were processed in order to make it clean, uniform, and consistent | Methods/statistical analysis |
| 2.2 | Describe whether variables were normalized and if so, how this was done | No normalization was performed |
| 2.3 | Provide details on the fraction of missing values (if any) and imputation methods | Proportion of missing data was low (<5% for variables entered into the random forest model, <10% for albumin, cholesterol and NT-proBNP) |
| 2.4 | Describe any feature selection processes applied | Feature selection were selected to include common and readily available clinical factors, echocardiographic parameters and laboratory |
| 2.5 | Identify and describe the process to handle outliers if any | NA |
| 2.6 | Describe whether class imbalance existed, and which method was applied to deal with it | Both datasets were split into derivation/validation cohorts, stratified by HF subgroups to ensure balanced distribution within the two cohorts and 70% served as derivation data |
| 3.1 | Explicitly define the goal of the analysis e.g., regression, classification, clustering | Methods/statistical analysis, variable importance analysis and investigation of non-linear associations |
| 3.2 | Identify the proper learning method used (e.g., supervised, reinforcement learning etc.) to address the problem | Methods/statistical analysis, supervised learning was utilized, specifically random survival forest was applied to investigate right-censored survival data. |
| 3.3 | Provide explicit details on the use of simpler, complex, or ensemble models | NA, no simpler models provide variable importance analysis and dependence/ partial dependency plot analysis in the setting of right censored survival data. Random forest, as an ensemble decision tree algorithm was utilized |
| 3.4 | Provide the comparison of complex models against simpler models if possible | NA |
| 3.5 | Define ensemble methods, if used | Methods/statistical analysis: A random forest is an ensemble method of a binary decision tree. |
| 3.6 | Provide details on whether the model is interpretable | Methods/statistical analysis, model interpretation by means of dependence and partial dependence plots |
| 4.1 | Provide a clear description of data used for training, validation, and testing | Methods/Study population/Derivation and validation datasets |
| 4.2 | Describe how the model parameters were optimized (e.g., optimization technique, number of model parameters etc.) | Methods/statistical analysis |
| 5.1 | Provide the metric(s) used to evaluate the performance of the model | Methods/statistical analysis and Results/Incremental prognostic information of machine learning derived thresholds: Cox regression (C-statistic) to evaluate performance of baseline model and extended model with |
| 5.2 | Define the prevalence of disease and the choice of the scoring rule used | NA |
| 5.3 | Report any methods used to balance the numbers of subjects in each class | NA |
| 5.4 | Discuss the risk associated to misclassification | NA |
| 6.1 | Consider sharing code or scripts on a public repository with appropriate copyright protection steps for further development and non-commercial use | A list of used packages for this analysis is presented in Supplementary Table 1 |
| 6.2 | Release a data dictionary with appropriate explanation of the variables | Methods/statistical analysis. Specific terms are explained |
| 6.3 | Document the version of all software and external libraries used | Supplementary Table 1 |
| 7.1 | Identify and report the relevant model assumptions and findings | Methods/statistical analysis |
| 7.2 | If well performing models were tested on a hold-out validation dataset, detail the data of that validation set with the same rigor as that of training dataset (see section 2 above) | Results/ Derivation and validation cohort baseline characteristics  All identified thresholds were validated on the validation cohort. |

**Supplementary Table 3**: Baseline laboratory characteristics of all patients with moderate and severe secondary tricuspid regurgitation.

| Characteristic | Overall, N = 4,868^1^ | moderate sTR, N = 3,359^1^ | severe sTR, N = 1,509^1^ | p-value^2^ |
| --- | --- | --- | --- | --- |
| ***Laboratory parameters*** |  |  |  |  |
| Hemoglobin, g/dl | 12.4 (10.8 - 13.8) | 12.5 (10.8 - 13.9) | 12.3 (10.8 - 13.6) | 0.11 |
| White blood cell count, G/l | 7.4 (6.0 - 9.1) | 7.4 (6.0 - 9.2) | 7.4 (6.0 - 9.0) | 0.2 |
| Platelets | 216.0 (173.0 - 267.0) | 218.0 (176.0 - 269.1) | 210.0 (168.0 - 259.0) | **<0.001** |
| Hs-CRP^4^ | 0.9 (0.3 - 2.6) | 0.8 (0.3 - 2.6) | 1.0 (0.4 - 2.6) | 0.005 |
| Creatinine, mg/dl | 1.1 (0.9 - 1.4) | 1.1 (0.9 - 1.4) | 1.2 (0.9 - 1.5) | **<0.001** |
| Glomerular filtration rate, ml/min/1.73m^2^ | 59 (43 - 74) | 60 (44 - 75) | 55 (41 - 69) | **<0.001** |
| Blood urea nitrogen, mg/dl | 20.6 (15.4 - 29.8) | 19.8 (15.0 - 27.8) | 22.6 (16.5 - 33.3) | **<0.001** |
| Albumin, g/l | 38.5 (34.2 - 41.5) | 38.8 (34.3 - 41.6) | 38.0 (33.9 - 41.2) | **0.003** |
| Bilirubin, mg/dl | 0.7 (0.5 - 1.0) | 0.7 (0.5 - 1.0) | 0.8 (0.6 - 1.3) | **<0.001** |
| Aspartate transaminase, U/l | 27 (21 - 37) | 27 (21 - 36) | 29 (22 - 40) | **<0.001** |
| Alanine transaminase, U/l | 24 (16 - 37) | 24 (16 - 36) | 24 (17 - 38) | 0.15 |
| Gamma-Glutamyl transferase, U/l | 53.0 (28.0 - 107.0) | 46.0 (25.0 - 90.5) | 75.0 (37.0 - 142.0) | **<0.001** |
| Total cholesterol, mg/dl | 153 (125 - 181) | 158 (129 - 185) | 142 (116 - 170) | **<0.001** |
| ^1^n (%); Median (IQR), ^2^Pearson's Chi-squared test, bold P values indicate statistical significance; Wilcoxon rank sum test, ^3^chornic obstructive pulmonary disease, ^4^high sensitivity C-reactive protein | | | | |

**Supplementary Table 4**: Baseline characteristics of all patients with severe secondary tricuspid regurgitation and stratified according to derivation and validation cohort.

| Characteristic | Derivation, N = 1,057^1^ | Validation, N = 452^1^ | P-value^2^ |
| --- | --- | --- | --- |
| Heart failure subtype |  |  | >0.9 |
| HFpEF | 497 (47%) | 213 (47%) |  |
| HFmrEF | 214 (20%) | 91 (20%) |  |
| HFrEF | 346 (33%) | 148 (33%) |  |
| Sex, male | 590 (56%) | 258 (57%) | 0.7 |
| Age, years | 74 (65 - 81) | 75 (67 - 81) | 0.2 |
| Body mass index, kg/m2 | 25.6 (22.5 - 29.4) | 24.8 (22.4 - 29.4) | 0.3 |
| Weight | 74 (65 - 87) | 73 (63 - 87) | 0.6 |
| Height | 170 (163 - 176) | 170 (164 - 177) | 0.4 |
| Hypertension | 557 (53%) | 244 (54%) | 0.6 |
| Hyperlipidemia | 276 (26%) | 117 (26%) | >0.9 |
| Diabete, type II | 223 (21%) | 109 (24%) | 0.2 |
| Coronary Artery Disease | 428 (40%) | 207 (46%) | 0.056 |
| Atrial fibrillation | 515 (49%) | 220 (49%) | >0.9 |
| COPD | 178 (17%) | 71 (16%) | 0.6 |
| Cerebral vascular disease | 188 (18%) | 70 (15%) | 0.3 |
| Peripheral vascular disease | 246 (23%) | 104 (23%) | >0.9 |
| Left ventricular end-diastolic diameter, mm | 46 (42 - 53) | 47 (42 - 53) | 0.7 |
| Left atrial diameter, mm | 65 (60 - 71) | 66 (60 - 71) | 0.6 |
| Right ventricular end-diastolic diameter, mm | 40 (35 - 44) | 40 (35 - 45) | 0.6 |
| Right atrial diameter, mm | 66 (60 - 72) | 66 (61 - 72) | 0.7 |
| Left ventricular dysfunction |  |  | >0.9 |
| Absent | 454 (43%) | 197 (44%) |  |
| Mild | 135 (13%) | 53 (12%) |  |
| Moderate | 122 (12%) | 54 (12%) |  |
| Severe | 346 (33%) | 148 (33%) |  |
| Right ventricular dysfunction |  |  | 0.6 |
| Mild | 494 (47%) | 224 (50%) |  |
| Moderate | 429 (41%) | 176 (39%) |  |
| Severe | 134 (13%) | 52 (12%) |  |
| Secondary mitral regurgitation |  |  | 0.5 |
| Mild | 100 (9.5%) | 34 (7.5%) |  |
| Moderate | 614 (58%) | 271 (60%) |  |
| Severe | 343 (32%) | 147 (33%) |  |
| Pulmonary artery pressure (mmHg) | 56 (48 - 70) | 59 (46 - 68) | 0.7 |
| PM leads present | 214 (20%) | 98 (22%) | 0.5 |
| Inferior vena cava diameter, mm | 24.0 (21.0 - 27.0) | 23.0 (20.0 - 28.0) | >0.9 |
| Hemoglobin, g/dl | 12.4 (10.8 - 13.6) | 12.2 (10.8 - 13.5) | 0.2 |
| White blood cell count, G/l | 7.4 (6.0 - 9.0) | 7.4 (5.8 - 8.8) | 0.7 |
| Platelets | 210.0 (168.0 - 256.0) | 209.5 (165.0 - 268.8) | 0.8 |
| Hs-CRP | 0.96 (0.34 - 2.48) | 1.04 (0.39 - 2.99) | 0.3 |
| Creatinine, mg/dl | 1.1 (0.9 - 1.5) | 1.2 (0.9 - 1.5) | 0.6 |
| Glomerular filtration rate, ml/min/1.73m^2^ | 55 (41 - 70) | 55 (41 - 69) | 0.6 |
| Blood urea nitrogen, mg/dl | 22.5 (16.6 - 33.7) | 23.3 (16.5 - 32.8) | >0.9 |
| Albumin, g/l | 38.0 (34.1 - 41.2) | 38.1 (33.6 - 41.2) | >0.9 |
| Bilirubin, mg/dl | 0.8 (0.6 - 1.3) | 0.8 (0.6 - 1.3) | 0.3 |
| Aspartate transaminase, U/l | 29 (22 - 41) | 29 (22 - 39) | 0.6 |
| Alanine transaminase, U/l | 25 (17 - 40) | 22 (17 - 33) | 0.061 |
| Gamma-Glutamyl transferase, U/l | 74.0 (37.0 - 135.0) | 77.0 (38.8 - 163.2) | 0.13 |
| Total cholesterol, mg/dl | 143 (118 - 170) | 140 (115 - 170) | 0.5 |
| Nt-proBNP, pg/ml | 3,704.0 (1,644.0 - 8,468.0) | 3,810.2 (1,874.5 - 7,673.0) | 0.7 |
| ^1^n (%); Median (IQR) | | | |
| ^2^Pearson's Chi-squared test; Wilcoxon rank sum test, bold P values indicate statistical significance | | | |

COPD = chronic obstructive pulmonary disease; hs-CRP = high sensitivity C-reactive protein, HFmrEF = heart failure with midrange ejection fraction, HFpEF = heart failure with preserved ejection fraction, HFrEF = heart failure with reduced ejection fraction, PM = pacemaker

**Supplementary Table 5:** Baseline characteristics of all patients with moderate secondary tricuspid regurgitation and stratified according to derivation and validation cohort.

| Characteristic | Derivation, N = 2,353^1^ | Validation, N = 1,006^1^ | P-value^2^ |
| --- | --- | --- | --- |
| Heart failure subtype |  |  | >0.9 |
| HFpEF | 1,283 (55%) | 549 (55%) |  |
| HFmrEF | 524 (22%) | 224 (22%) |  |
| HFrEF | 546 (23%) | 233 (23%) |  |
| Sex, male | 1,386 (59%) | 611 (61%) | 0.3 |
| Age, years | 73 (66 - 80) | 73 (65 - 80) | 0.3 |
| Body mass index, kg/m2 | 26.6 (24.2 - 29.4) | 26.6 (24.2 - 29.7) | 0.6 |
| Weight | 79 (69 - 89) | 78 (70 - 90) | 0.4 |
| Height | 170 (163 - 177) | 170 (164 - 177) | 0.4 |
| Hypertension | 1,463 (62%) | 631 (63%) | 0.8 |
| Hyperlipidemia | 768 (33%) | 316 (31%) | 0.5 |
| Diabete, type II | 587 (25%) | 225 (22%) | 0.11 |
| Coronary Artery Disease | 1,116 (47%) | 456 (45%) | 0.3 |
| Atrial fibrillation | 944 (40%) | 426 (42%) | 0.2 |
| COPD | 328 (14%) | 140 (14%) | >0.9 |
| Cerebral vascular disease | 465 (20%) | 195 (19%) | 0.8 |
| Peripheral vascular disease | 559 (24%) | 247 (25%) | 0.6 |
| Left ventricular end-diastolic diameter, mm | 47 (42 - 52) | 47 (43 - 52) | 0.2 |
| Left atrial diameter, mm | 61 (56 - 67) | 61 (57 - 67) | 0.15 |
| Right ventricular end-diastolic diameter, mm | 35 (31 - 39) | 35 (31 - 39) | >0.9 |
| Right atrial diameter, mm | 60 (55 - 65) | 60 (55 - 65) | 0.5 |
| Left ventricular dysfunction |  |  | >0.9 |
| Absent | 1,170 (50%) | 504 (50%) |  |
| Mild | 334 (14%) | 141 (14%) |  |
| Moderate | 303 (13%) | 128 (13%) |  |
| Severe | 546 (23%) | 233 (23%) |  |
| Right ventricular dysfunction |  |  | 0.14 |
| Mild | 1,812 (77%) | 782 (78%) |  |
| Moderate | 461 (20%) | 178 (18%) |  |
| Severe | 80 (3.4%) | 46 (4.6%) |  |
| Secondary mitral regurgitation |  |  | 0.7 |
| Mild | 312 (13%) | 145 (14%) |  |
| Moderate | 1,698 (72%) | 716 (71%) |  |
| Severe | 343 (15%) | 145 (14%) |  |
| Pulmonary artery pressure (mmHg) | 48 (41 - 59) | 48 (41 - 59) | 0.7 |
| PM leads present | 328 (14%) | 127 (13%) | 0.3 |
| Inferior vena cava diameter, mm | 21.0 (18.0 - 24.0) | 20.0 (17.0 - 23.0) | 0.14 |
| Hemoglobin, g/dl | 12.5 (10.8 - 13.8) | 12.5 (10.8 - 13.9) | 0.8 |
| White blood cell count, G/l | 7.4 (6.1 - 9.2) | 7.4 (6.0 - 9.2) | 0.6 |
| Platelets | 219.0 (179.0 - 269.0) | 216.0 (171.0 - 269.8) | 0.2 |
| Hs-CRP | 0.8 (0.3 - 2.6) | 0.9 (0.3 - 2.6) | >0.9 |
| Creatinine, mg/dl | 1.1 (0.9 - 1.4) | 1.0 (0.9 - 1.4) | 0.026 |
| Glomerular filtration rate, ml/min/1.73m^2^ | 60 (44 - 75) | 62 (45 - 77) | 0.017 |
| Blood urea nitrogen, mg/dl | 19.9 (15.1 - 28.5) | 19.7 (14.8 - 26.5) | 0.13 |
| Albumin, g/l | 38.7 (34.6 - 41.6) | 38.8 (34.0 - 41.7) | 0.8 |
| Bilirubin, mg/dl | 0.7 (0.5 - 1.0) | 0.7 (0.5 - 1.0) | 0.8 |
| Aspartate transaminase, U/l | 26 (21 - 36) | 27 (21 - 36) | >0.9 |
| Alanine transaminase, U/l | 24 (16 - 36) | 23 (16 - 36) | 0.5 |
| Gamma-Glutamyl transferase, U/l | 48.0 (26.0 - 95.0) | 43.5 (24.0 - 81.0) | **0.022** |
| Total cholesterol, mg/dl | 158 (129 - 185) | 158 (130 - 187) | 0.5 |
| Nt-proBNP, pg/ml | 2,242.9 (878.0 - 5,083.0) | 2,252.0 (856.6 - 4,911.8) | 0.8 |
| ^1^n (%); Median (IQR) | | | |
| ^2^Pearson's Chi-squared test; Wilcoxon rank sum test, bold P values indicate statistical significance | | | |

COPD = chronic obstructive pulmonary disease; hs-CRP = high sensitivity C-reactive protein, HFmrEF = heart failure with midrange ejection fraction, HFpEF = heart failure with preserved ejection fraction, HFrEF = heart failure with reduced ejection fraction, PM = pacemaker

**Table 6:** Univariable and adjusted Cox analysis for derivation cohort.

| **Moderate sTR** | **Derivation (n = 2353)** | |  |
| --- | --- | --- | --- |
|  | **Hazard ratio (95% CI)** | **p- value†** |  |
| **Univariable Cox analysis** |  |  |  |
| Albumin ≥ 40 g/L | 0.43 (0.37-0.50) | **<0.001** |  |
| NT-proBNP ≥ 4000 pg/ml | 2.45 (2.15-2.8) | **<0.001** |  |
| Hemoglobin ≥ 13 g/dl | 0.48 (0.42-0.55) | **<0.001** |  |
| Hs-CRP^1^ ≥ 1.0 mg/dl | 1.95 (1.71-2.22) | **<0.001** |  |
| GFR ≥ 60 ml/min/1.73m^2^ | 0.55 (0.48-0.63) | **<0.001** |  |
| **Clinical risk factor adjustd Cox analysis*** |  |  |  |
| Albumin ≥ 40 g/L | 0.50 (0.43-0.58) | **<0.001** |  |
| NT-proBNP ≥ 4000 pg/ml | 1.97 (1.69-2.30) | **<0.001** |  |
| Hemoglobin ≥ 13 g/dl | 0.50 (0.43-0.58) | **<0.001** |  |
| Hs-CRP^1^ ≥ 1.0 mg/dl | 1.74 (1.52-1.98) | **<0.001** |  |
| GFR ≥ 60 ml/min/1.73m^2^ | 0.58 (0.50-0.67) | **<0.001** |  |
| **Severe sTR** | **Derivation (n = 1057)** |  |  |
| **Univariable Cox analysis** |  |  |  |
| Albumin ≥ 40 g/L | 0.55 (0.45-0.68) | **<0.001** |  |
| NT-proBNP ≥ 4000 pg/ml | 2.27 (1.89-2.72) | **<0.001** |  |
| Hemoglobin ≥ 13 g/dl | 0.61 (0.50-0.73) | **<0.001** |  |
| Hs-CRP^1^ ≥ 1.0 mg/dl | 1.75 (1.46-2.09) | **<0.001** |  |
| GFR ≥ 60 ml/min/1.73m^2^ | 0.57 (0.47-0.70) | **<0.001** |  |
| **Clinical risk factor adjustd Cox analysis*** |  |  |  |
| Albumin ≥ 40 g/L | 0.63 (0.51-0.78) | **<0.001** |  |
| NT-proBNP ≥ 4000 pg/ml | 1.86 (1.50-2.29) | **<0.001** |  |
| Hemoglobin ≥ 13 g/dl | 0.67 (0.55-0.83) | **<0.001** |  |
| Hs-CRP^1^ ≥ 1.0 mg/dl | 1.59 (1.33-1.92) | **<0.001** |  |
| GFR ≥ 60 ml/min/1.73m^2^ | 0.63 (0.52-0.78) | **<0.001** |  |
| *adjusted for sex, age, history of ischemic heart disease, serum creatinine, LV end-diastolic diameter, left ventricular function and right ventricular function | | | |

^1^high sensitivity C-reactive protein

†bold P values indicate statistical significance

**Table 7:** Cox regression analysis in validation cohort for adverse feature subgroups

| **Moderate sTR** | **Derivation** | |
| --- | --- | --- |
|  | **Hazard ratio (95% CI)** | **p-value†** |
| 0 or 1 adverse features (Reference) |  |  |
| 2 or 3 adverse features | 2.30 (1.90-2.79) | **<0.001** |
| 4 or 5 adverse features | 4.46 (3.69-5.40) | **<0.001** |
| **Severe sTR** |  |  |
| 0 or 1 adverse features (Reference) |  |  |
| 2 or 3 adverse features | 2.60 (1.90-3.57) | **<0.001** |
| 4 or 5 adverse features | 4.44 (3.26-6.06) | **<0.001** |

†bold P values indicate statistical significance

**Supplementary Table 8:** Improved prognostic information in validation datasets with machine learning identified variables. Model 1 represents the baseline clinical risk factor model encompassing age, gender, ischemic heart disease, serum creatinine levels, left ventricular end-diastolic diameter, left ventricular and right ventricular function and Model 2 additionally incorporates the machine learning derived variables.

| **Moderate sTR** | **Model** | **C-statistic** |
| --- | --- | --- |
|  | Model 1 | 0.645 |
|  | Model 2 | 0.713 |
| **Severe sTR** |  |  |
|  | Model 1 | 0.643 |
|  | Model 2 | 0.700 |

**Supplementary Figure 1: Dependency plots of the five most predictive variables in the moderate sTR RSF model from the derivation cohort.**

The five most predictive variables were (A) albumin, (B) NT-proBNP, (C) hemoglobin, (D) C-reactive protein (hs-CRP) and (E) glomerular filtration rate (GFR). Variable dependency plots depict the predicted mortality at 6 years in relation to the selected variable. Blue line indicates the overall trend with a LOESS curve, while grey areas indicate the 95% confidence interval. Each point is a patient, bronze color indicates an event, while grey points are patients alive at 6 years.


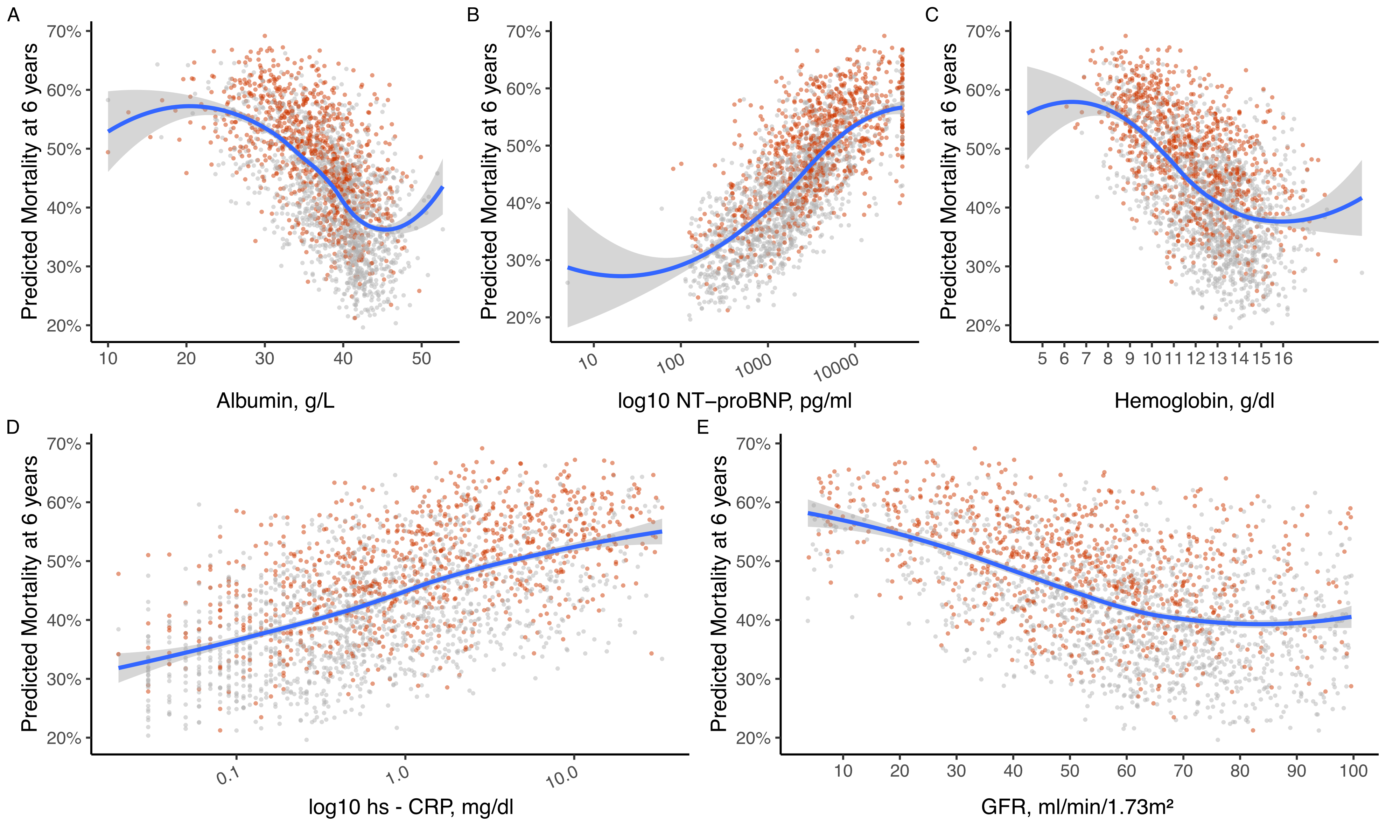


**Supplementary Figure 2: Dependency plots of the five most predictive variables in the severe sTR RSF model from the derivation cohort.**

The five most predictive variables were (A) NT-proBNP, (B) albumin(C) glomerular filtration rate (GFR), (D) C-reactive protein (hs-CRP) and (E) hemoglobin. Variable dependency plots depict the predicted mortality at 6 years in relation to the selected variable. Blue line indicates the overall trend with a LOESS curve, while grey areas indicate the 95% confidence interval. Each point is a patient, bronze color indicates an event, while grey points are alive at 6 years.


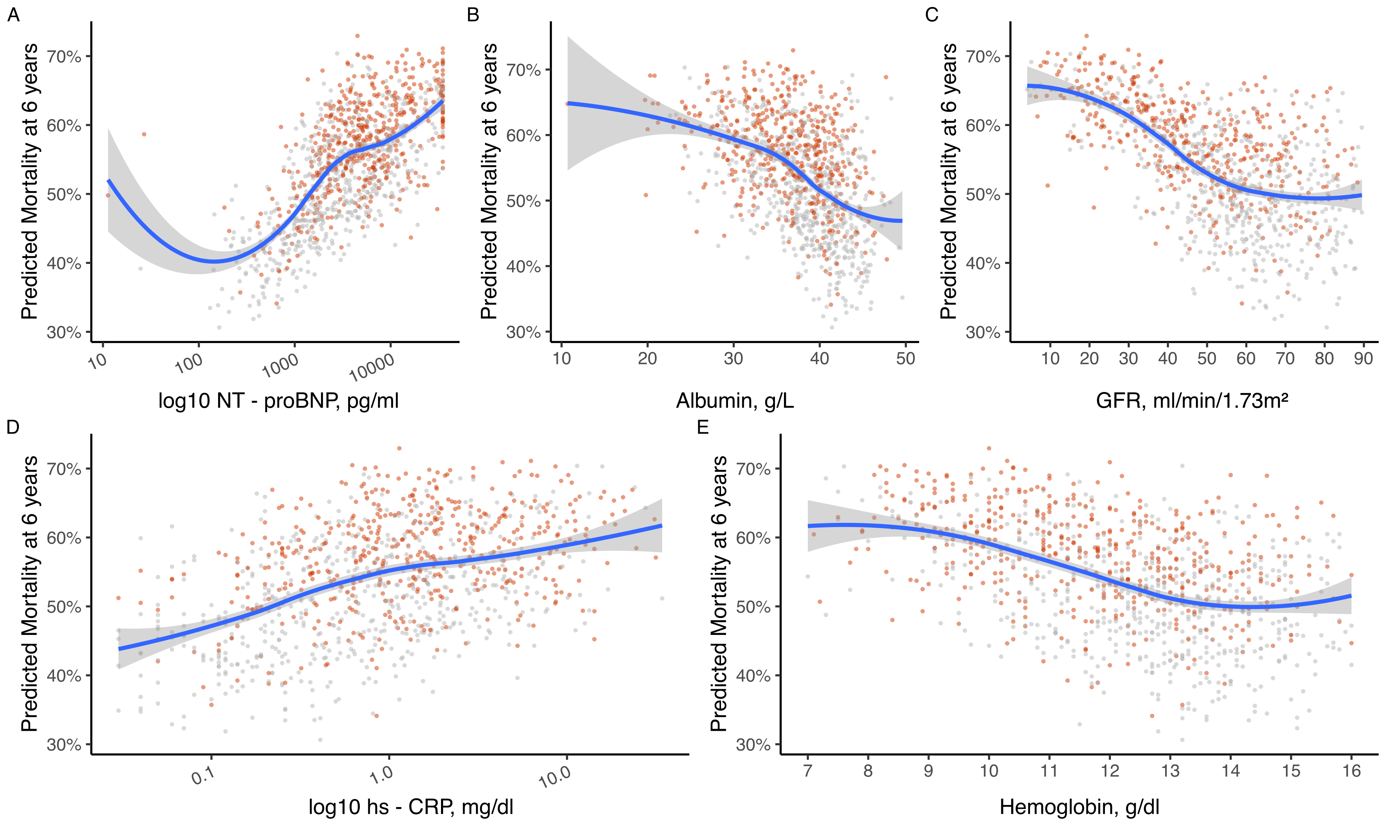


**Supplementary Figure 3: Histograms of the five most predictive variables in the moderate sTR RSF model from the derivation cohort.**

Distribution of the five most predictive variables (A) albumin, (B) NT-proBNP, (C) hemoglobin, (D) C-reactive protein (hs-CRP) and (E) glomerular filtration rate (GFR).


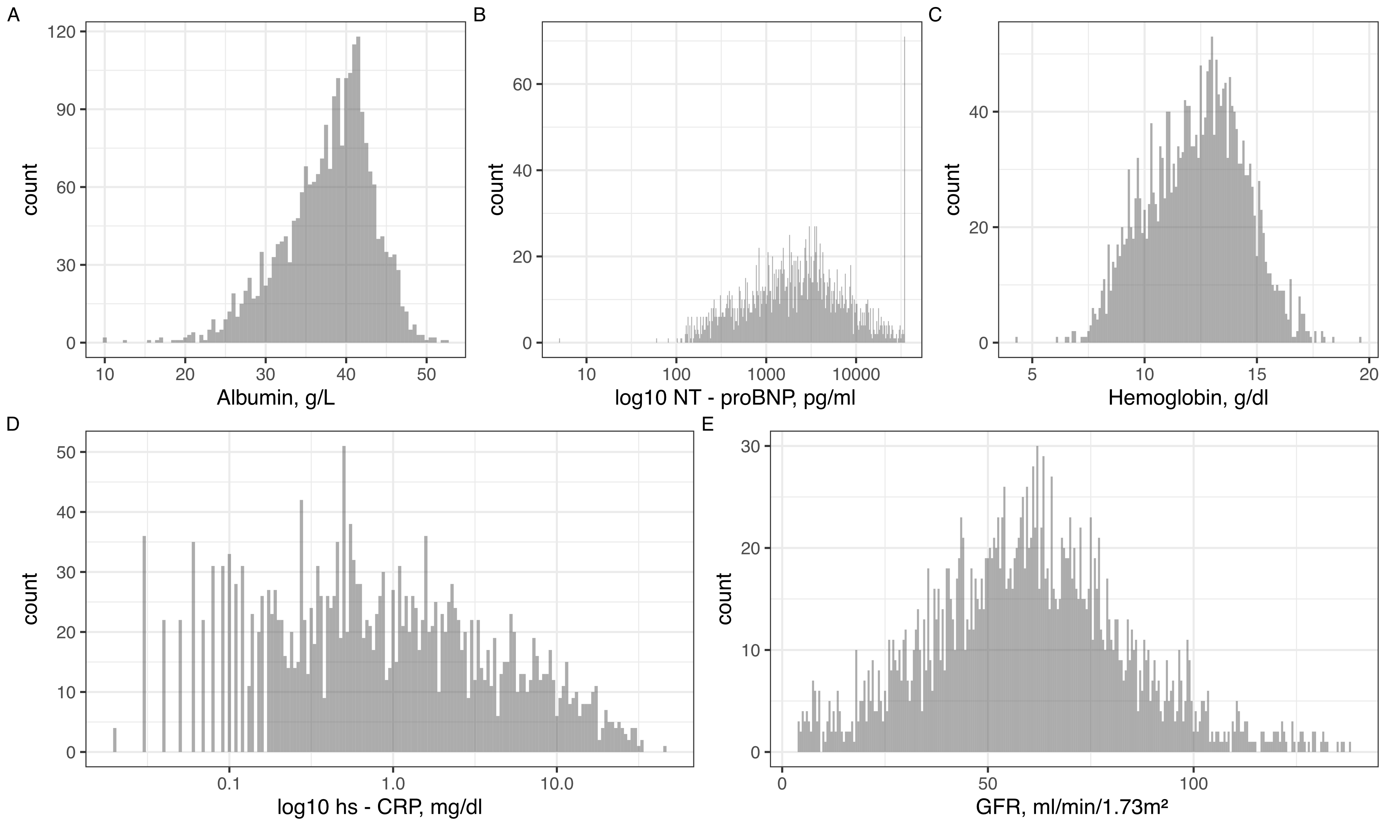


**Supplementary Figure 4: Histograms of the five most predictive variables in the severe sTR RSF model from the derivation cohort.**

Distribution of the five most predictive variables (A) NT-proBNP, (B) albumin(C) glomerular filtration rate (GFR)., (D) C-reactive protein (hs-CRP) and (E) hemoglobin.


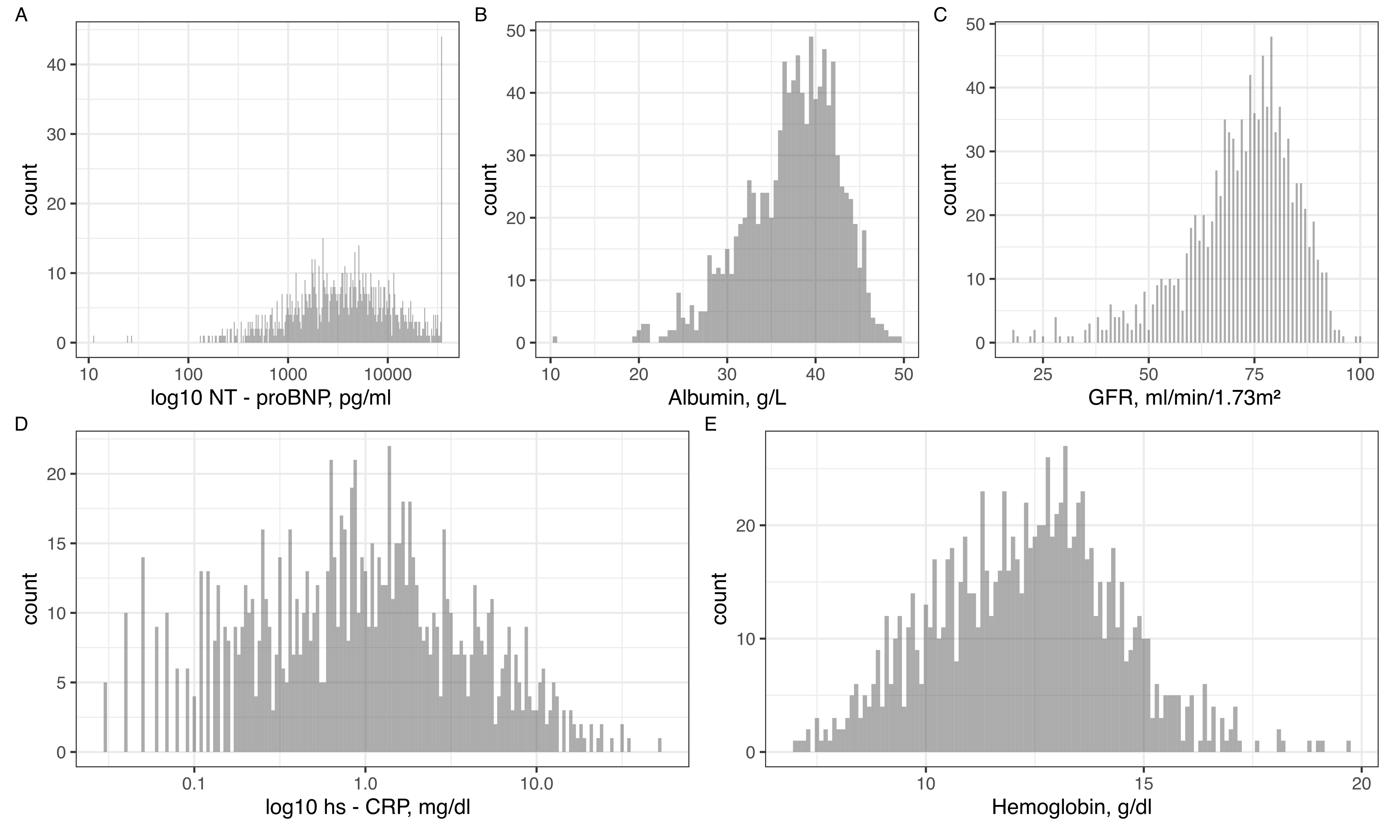


**Supplementary Figure 5: Kaplan Meier analysis fer derived cut-offs in moderate sTR.** All cutoffs are presented in the respective derivation (Plot A, C, E, G and I) and validation cohort (Plots B, D, F, H and J).


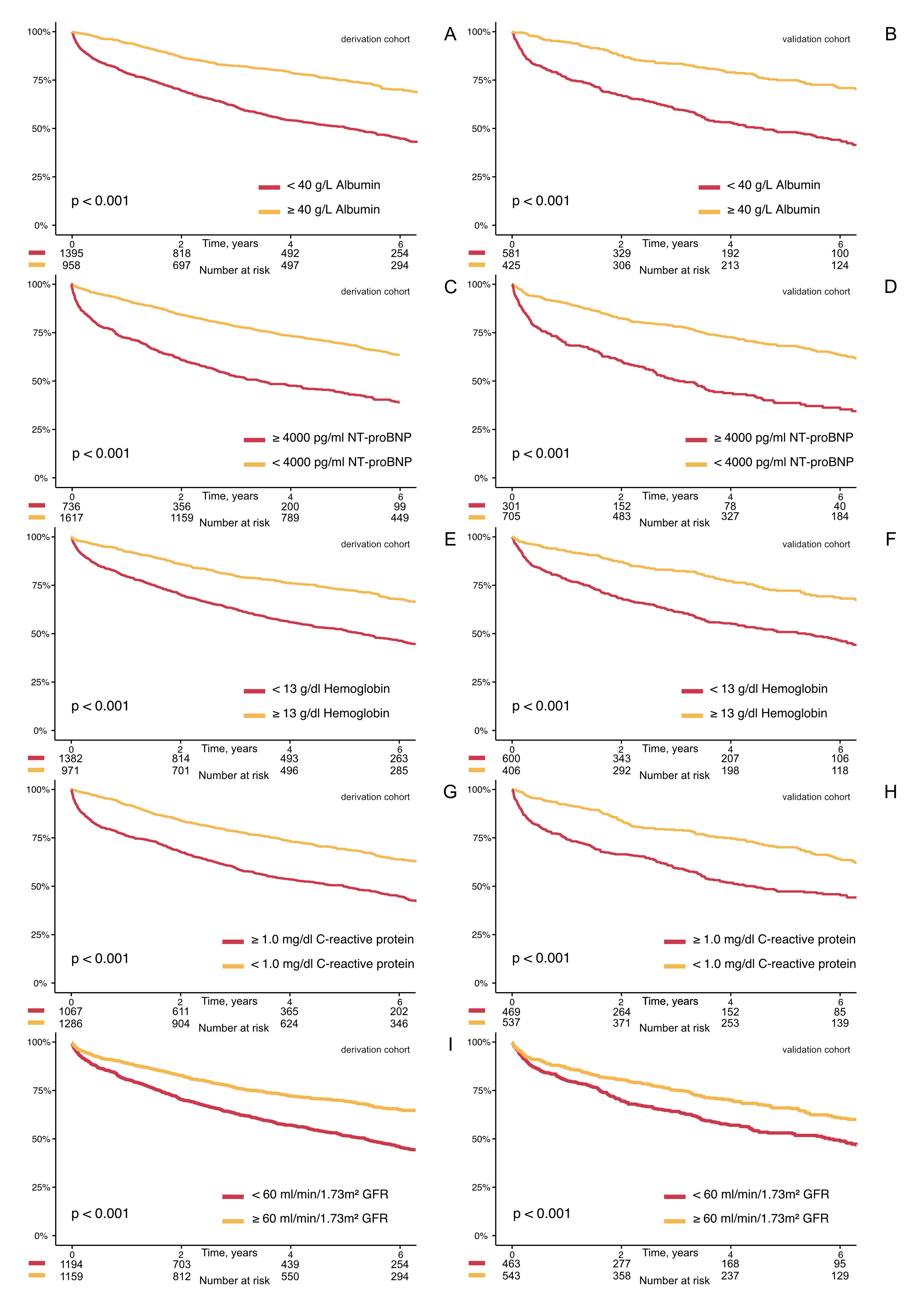


**Supplementary Figure 6: Kaplan Meier analysis fer derived cut-offs in severe sTR.** All cutoffs are presented in the respective derivation (Plot A, C, E, G and I) and validation cohort (Plots B, D, F, H and J).


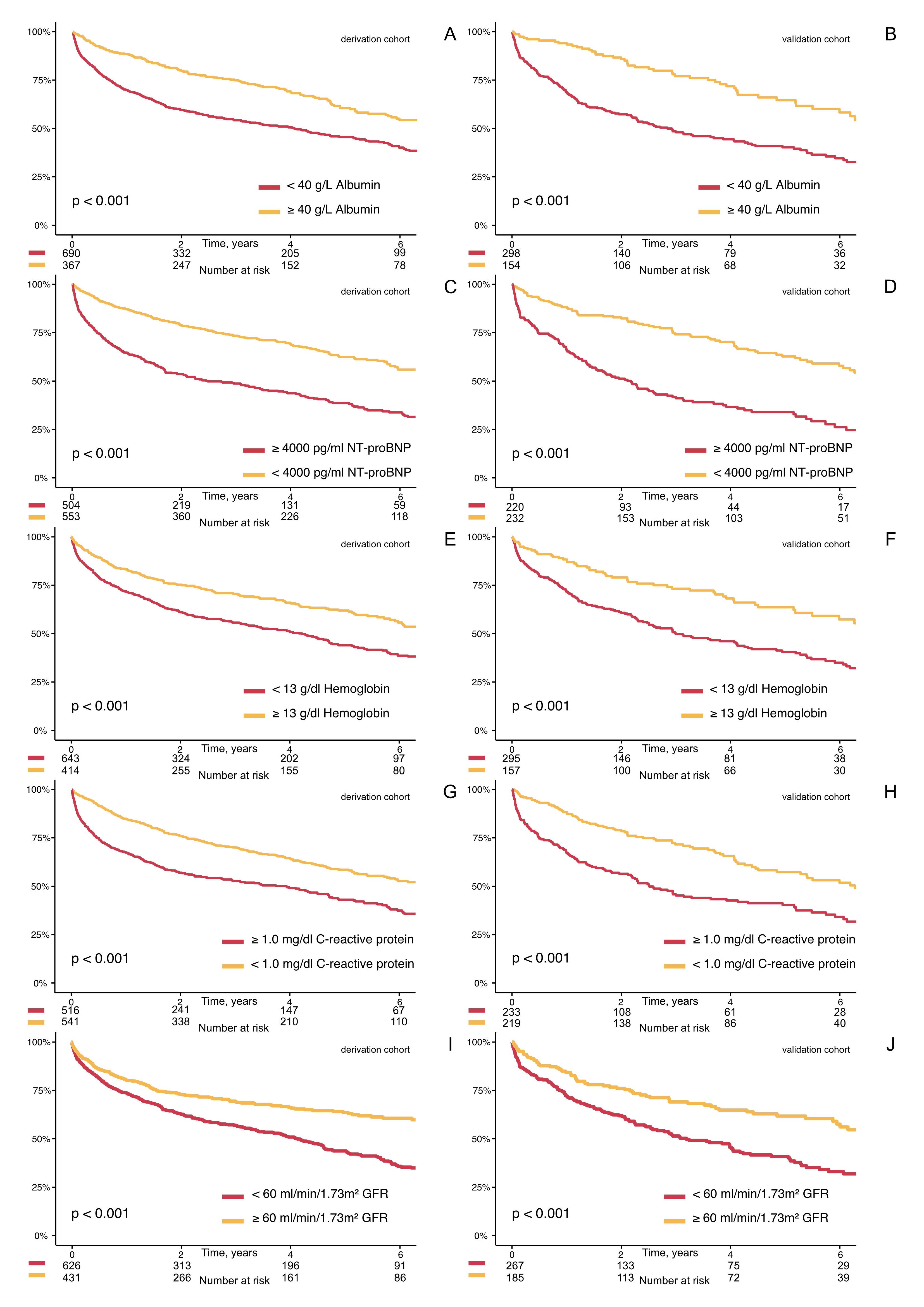


**References:**

1. Waljee AK, Mukherjee A, Singal AG*, et al.* Comparison of imputation methods for missing laboratory data in medicine. *BMJ Open* 2013;**3**. doi: 10.1136/bmjopen-2013-002847

2. Sengupta PP, Shrestha S, Berthon B*, et al.* Proposed Requirements for Cardiovascular Imaging-Related Machine Learning Evaluation (PRIME): A Checklist: Reviewed by the American College of Cardiology Healthcare Innovation Council. *JACC Cardiovasc Imaging* 2020;**13**:2017-2035. doi: 10.1016/j.jcmg.2020.07.015

3. Ambale-Venkatesh B, Yang X, Wu CO*, et al.* Cardiovascular Event Prediction by Machine Learning: The Multi-Ethnic Study of Atherosclerosis. *Circ Res* 2017;**121**:1092-1101. doi: 10.1161/CIRCRESAHA.117.311312

4. Ishwaran H, Kogalur UB, Blackstone EH, Lauer MS. Random Survival Forests. *Annals of Applied Statistics* 2008;**2**:841-860. doi: 10.1214/08-Aoas169
